# Genetic analysis of four cases of Poirier Bienvenu neurodevelopmental syndrome associated with *CSNK2B* variant

**DOI:** 10.1186/s12920-025-02132-5

**Published:** 2025-04-10

**Authors:** Liu Yang, Daoqi Mei, Yanping Liu, Li Gao

**Affiliations:** 1 Department of Pediatrics, Henan Provincial People’s Hospital, Zhengzhou University People’s Hospital, Zhengzhou, Henan 450003 China; 2 Department of Neurology, Children’s Hospital of Soochow University, Suzhou, Jiangshu 215127 China

**Keywords:** Poirier-Bienvenu neurodevelopmental syndrome, *CSNK2B*, Whole exome sequencing, Epilepsy, cDNA sequencing

## Abstract

**Background:**

CSNK2B deficiency underlies the pathogenesis of Poirier-Bienvenu neurodevelopmental syndrome (POBINDS). In this study, we present four cases of pediatric seizures caused by de novo variants in *CSNK2B*, with the aim to reinforce the clinical and variant data pertaining to early genetic factors associated with epilepsy.

**Methods:**

Trio whole exome sequencing were used to detect variants in the proband and her family members, and bioinformatics annotation was performed for the variant. Sanger sequencing and *CSNK2B* cDNA sequencing were employed to ascertain the carrier status of additional family members and evaluate the potential impact of variants on splicing.

**Results:**

All four cases presented with epilepsy as the initial manifestation, accompanied by global developmental delay, particularly in language and motor developmental delay. Cases 1, 3 and 4 exhibited full-scale tonic-clonic seizures, while case 2 displayed myoclonic and typical absence seizures. Furthermore, case 2 demonstrated delayed growth and development compared to age-matched peers. No abnormality was detected in the head magnetic resonance imaging (MRI). Genetic analysis revealed novel heterozygous variants in the *CSNK2B* gene in all four cases, including c.175 + 1G > A, c.73-2A > G, c.291 + 1G > A and c.481delA. In case 2, reverse transcription analysis of *CSNK2B* mRNA revealed the retention of the 3’ end sequence of Intron 2 and deletion of the 5’ end sequence of Exon 3. In treatment, four case received a combination of one to three types of antiseizure medication and rehabilitation training individually. Case 1 continued to experience seizures to varying degrees, while cases 2–4 demonstrated effective seizure control. Overall motor and intellectual development improved in all four cases, however, there was slow recovery in language function.

**Conclusion:**

This study elucidates the molecular etiology of epilepsy in four cases with POBINDS and expands the mutational spectrum of pathogenic variants in the *CSNK2B*, highlighting their impact on splicing. The highly genetic heterogeneous phenotype of POBINDS relies on the detection of pathogenic variants in *CSNK2B*. Conventional antiseizure medication effectively control seizures, while rehabilitation treatment can significantly improve intelligence and motor function to varying degrees; however, language recovery tends to be relatively slow.

**Supplementary Information:**

The online version contains supplementary material available at 10.1186/s12920-025-02132-5.

## Background

Poirier Bienvenu neurodevelopmental syndrome (POBINDS, OMIM: # 618732) is a rare autosomal dominant inherited disease. In 2017, Poirier et al. [1]. reported for the first time that splicing site variants in the *CSNK2B* gene in two cases could lead to intellectual impairment with or without myoclonic epilepsy. POBINDS are clinically characterized by early-onset seizures and developmental disorders. However, due to the high heterogeneity of individual phenotypes, early clinical diagnosis is difficult, mainly based on the detection of heterozygous pathogenic variants in the *CSNK2B* gene. The majority of cases reported neurodevelopmental disorders (90%) and epilepsy (82%), with onset predominantly occurring within the first six months of life (69%) [2]. Delays were observed across multiple developmental domains, with language and cognitive impairments being most prominent. While motor developmental delays were common, nearly all cases retained ambulatory capabilities. In contrast, one in five cases exhibited no language ability, and over two-thirds demonstrated cognitive impairments ranging from mild to severe [2, 3].

*CSNK2B* gene encodes protein kinase CK2β, The latter belongs to the eosinophilic serine/threonine kinase subfamily CK2, which can participate in cell apoptosis, proliferation, and DNA damage responses, as well as phosphorylation of various substrates in multiple signaling pathways including NF-kB, PTEN/PI3K/Akt, and Wnt/b - catenin biological processes [1, 4]. CK2 is highly expressed in the brain, particularly in neurons and neuroepithelial cells [5]. where it is involved in neuronal development and plays a critical role in synaptic transmission [6]. CK2β Regarded as a regulatory subunit, its sequence is highly conserved. Previous studies have shown that if the regulatory subunit is overexpressed, CK2 can be formed β the trend of dimers is that dimers are a prerequisite for the formation and correct function of the entire enzyme. This may be significantly correlated with epilepsy and intellectual disability [3].

The mechanisms by which alterations in CSNK2B contribute to neurodevelopmental disorders (NDD) and seizures remain unclear. However, several potential explanations can be proposed. Loss-of-function (LoF) variants likely indicate haploinsufficiency, whereas missense variants may lead to a reduction in the functionality or stability of the CSNK2B protein complexes [7–9]. Furthermore, it was observed that inhibition of CK2 activity resulted in a significant reduction in the phosphorylation level of the Kv3.1 channel, thereby altering its potential. This phenomenon may be intricately linked to the pathogenesis of epilepsy [7]. Numerous studies have investigated the relationship between genotype and phenotype, thereby broadening our understanding of the genotype-phenotype spectrum. However, this relationship remains not yet to fully elucidated [2, 3, 7, 10].

This study reports on four POBINDS cases harboring CSNK2B gene variants. The clinical manifestations included seizures and developmental disabilities of varying severity. Furthermore, we conducted an analysis of the clinical phenotypes associated with POBINDS to provide a foundation for further investigation into the correlation between genotype and phenotype.

## Methods

### Patients selection

From March 2019 to June 2023, Henan Provincial People’s Hospital treated 4 cases of POBINDS, including 3 females and 1 male, with an onset age of 5 months to 5 years old. All 4 cases met the diagnostic criteria for POBINDS: (1) had a history of intellectual disability or convulsions; (2) Peripheral blood gene testing revealed pathogenic variants in the *CSNK2B* gene; (3) The age of onset is ≤ 14 years old. Exclusion criteria: Genetic testing of peripheral blood indicated that alternative gene variants were responsible for the epileptic encephalopathy. This study was approved by the Ethics Committee of Henan Provincial People’s Hospital (Ethics Approval No. 2019 Lunshen No. 15), and all guardians of the cases signed informed consent forms.

### Clinical data collection

Clinical data of 4 pediatric cases were collected, including gender, age, age of onset, clinical phenotype of epilepsy, family history, blood routine, blood ammonia, lactate, homocysteine, liver function, kidney function, thyroid function, screening of hematuria genetic metabolism, Gesell fertility scale, growth and development, 24-hour video electroencephalogram, head magnetic resonance imaging, etc. Regular outpatient follow-up, with follow-up up to June 2023, recording the incidence and medication adjustments.

### Trio whole exome sequencing

Trio-WES and data analysis were completed by Beijing Chigene Translational Medicine Research Center Co., Ltd, 100,875, Beijing. WES used The xGen Exome Research Panel v2.0 (IDT, USA) full exome capture chip to construct an exome library. Perform high-throughput sequencing using the NovaSeq6000 series sequencer (Illumina, USA). The sequencing process and fastq data generation, removal of joints, and low-quality reads cleaning and quality inspection are carried out using the system provided by the manufacturer, following the recommended standard procedures. Use BWA software to align the quality control sequence with the reference genome sequence of GRCh37/hg19. Perform gene variant analysis using GATK software. Complete variant annotation based on population genetic variant frequency databases (1,000 genomes, dbSNP, ESP, ExAC, and gnomAD), disease databases (HGMD, OMIM, Clinvar), and protein hazard and nucleic acid conservation prediction software (Provean, Sift, Revel, GERP, phyloP, phasCons, MaxEntScan, dbscSNV, GTAG). Then, refer to the 2015 ACMG (American College of Medical Genetics and Genomics) [11] and the 2018 Clinical Genetic Testing Report Specification [12] to interpret genetic variants. The *CSNK2B* gene reference transcript uses RefSeq: NM:001320.7. The sanger validation primers are designed for the variants (Supplementary Table 1).

### cDNA analysis

Blood samples were collected from the proband and anticoagulated with EDTA. Then, RNA was extracted using the Blood RNA Extraction Kit (Tiangen Biochemical Technology Co., Ltd., Beijing), followed by reverse transcription with random hexamers (Vazyme, China) to obtain cDNA. Based on *CSNK2B* (NM:001320.7→ NP_001311.3), c.73–2 A > G specific primers were designed, WDR45 As control (Supplementary Table 2). PCR was performed to amplify the target sequences, followed by 1.0% agarose gel electrophoresis and Sanger sequencing using the ABI3730 sequencer for verification. The results were analyzed using Chromas software.

## Results

### Clinical data

Basic information case 1, female, 3 years old. case 2, female, 6.1 years old. case 3, male, 4.3 years old. case 4, 5 years old (Table 1).

**Table 1:** Genotypes and clinical phenotypes of four children with epilepsy caused by *CNSK2B* gene variations

	Gender	Epilepsy onset time	Age (Year)	Clinical phenotype of epilepsy	Intelligent development	Motor development	Language development	Growth and development	Characteristics of video electroencephalogram	Variations	Pathogenic	Treatment	follow-up
1	female	0.9	3	GTCS	moderate	mild	moderate	normal	Bilateral frontal pole, frontal and anterior temporal region sharp slow wave emission	c.175+1G> A	PVS1+PS2+PM2_Supporting	LEV, OXC, CZP	Still having 2-3 episodes per day
2	female	3.9	1	JME	mild	normal	mild	backward	Widespread spike slow wave bursts	c.73–2 A> G	PVS1+PS2+PM2_Supporting	VPA	No attack
				AS									
3	male	0.4	4.3	GTCS	Moderate	mild	moderate	normal	Widespread spike and slow wave distribution mainly in bilateral temporal regions	c.291+1G> A	PVS1+PS2+PM2_Supporting	VPA	No attack
4	female	1	5	GTCS	mild	normal	moderate	normal	Bilateral frontal pole frontal and frontal line area spike slow wave emission	c.481delA	PVS1+PS2+PM2_Supporting	VPA	No attack

*Abbreviations*: GTCS generalized tonic-clonic seizure, JME positive myoclonic seizure, AS absence seizure, LEV levetiracetam, OXC oxcarbazepine, CZP clonazepam, VPA sodium valproate

### Epilepsy situation

Epilepsy was the first symptom in all four cases. Cases 1, 3, and 4 were generalized tonic-clonic seizures, while case 2 were myoclonic seizures and typical absence seizures (Table 1).

Case 1 experienced his first seizure at 9 months of age, over the following three months, the case experienced intermittent seizures. The seizure was characterized by upturned eyes, cyanotic lips, clenched fists, flexion and rigidity of both upper limbs, and rigidity of both lower limbs, accompanied by loss of consciousness. Each episode lasted approximately one minute. Seven hours later, a second seizure occurred when the case’s body temperature reached 38.5℃, raising concerns about febrile convulsions. Levetiracetam was initially prescribed at a dose of 10 mg/kg. Due to inadequate symptom control, the dosage was gradually increased to 20 mg/kg. Following genetic testing, sodium valproate oral solution was introduced into the treatment regimen. However, subsequent laboratory tests revealed elevated levels of alanine aminotransferase and aspartate aminotransferase, leading to the discontinuation of sodium valproate. Ultimately, oxcarbazepine and clonazepam were added to the treatment plan.

Case 2, at the age of three years, experienced seizures without an identifiable precipitating factor. During these episodes, the case exhibited a fixed gaze, was unresponsive to verbal stimuli, and demonstrated limb tremors lasting from 2 to 20 s. Despite initial improvement with pharmacological intervention, similar episodes recurred following dose reduction. Case 3 experienced unprovoked convulsions characterized by loss of consciousness, unresponsiveness to stimuli, fixed gaze, cyanosis of the lips, generalized rigidity, and limb shaking in 5-month-old. The seizures have occurred intermittently at a frequency of 1–2 times every two weeks and continue to occur with the same symptoms. Occasionally, these episodes are accompanied by a dull expression. Case 4 experienced a total of three seizures over a five-year period. Each episode was characterized by loss of consciousness, falling, upward deviation of the eyes, trismus, cyanosis of the lips, and limb rigidity. These episodes resolved spontaneously within approximately three minutes.

### Personal and family history

The parents of the 4 cases were non-consanguineous, and none of their parents or brothers had a history of epilepsy. Four cases with milestone developmental delay (Supplementary Fig. 1). In addition, Case 3 exhibited a dull facial expression and micropenis. Case 4 displayed irregular white spots on the left thigh. Case 2 presented with mild proportionate short stature with a Height Standard Deviation Score of − 3SD. During the most recent follow-up uterine ultrasound, Case 2 was found to have multiple small follicles in both ovaries and a small amount of fluid accumulation in the pelvic cavity.

### Auxiliary examinations

**Fig. 1:**
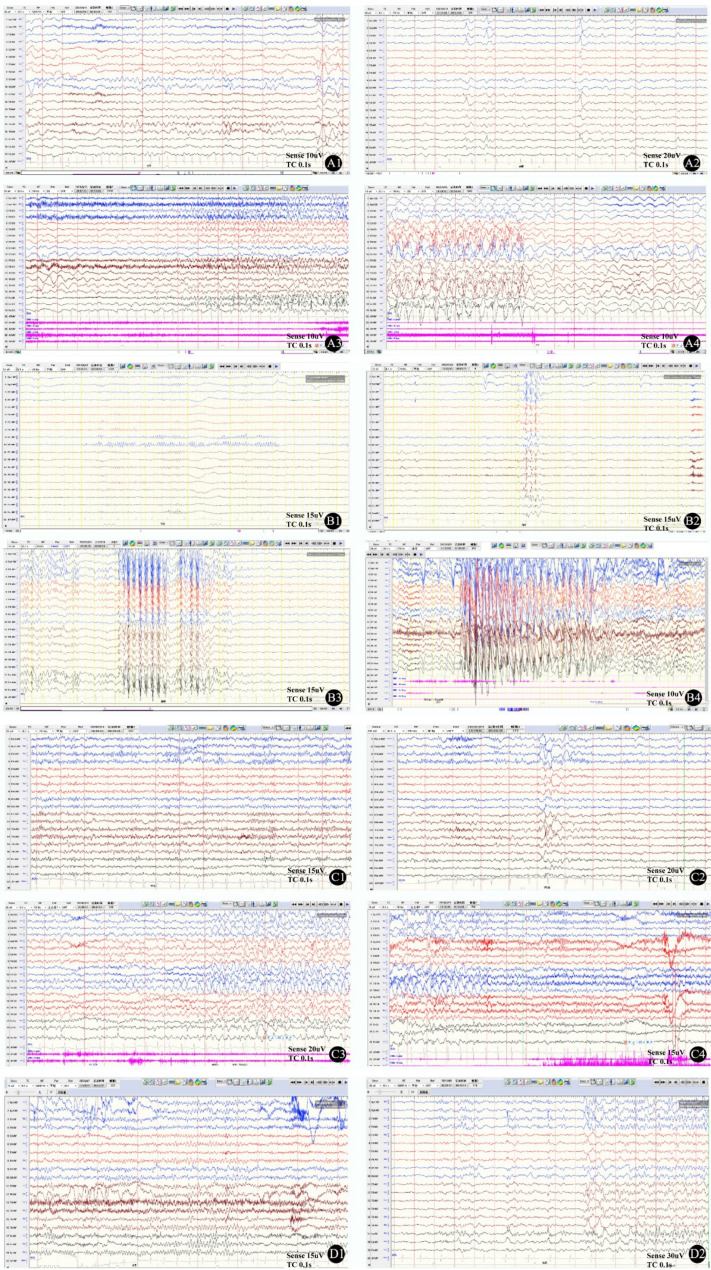
EGG images of 4 affected individuals. Case 1: **A1** Background bilateral occipital region 5–6 Hz low to medium wave amplitude θ Rhythm; During the interval between **A2** attacks, both sides of the frontal pole, frontal, and anterior temporal regions showed synchronous or asynchronous distribution of high amplitude sharp slow waves; Monitoring of bilateral anterior head initial focal secondary generalized tonic-clonic seizures during **A3**-**A4** seizure period **B**; Case 2: **B1** Background bilateral occipital region 8 Hz low to medium amplitude α Rhythm; Widespread mid to high amplitude spike slow wave bursts during the interval between **B2**-**B3** attacks; Typical absence episodes detected during **B4** episodes; Case 3:**C1** Background bilateral occipital region 8–9 Hz low to middle wave amplitude α Rhythm; During the interval between **C2** attacks, Bilateral temporal and extensive regions showed spike slow waves; Monitoring of right hemisphere initial focal secondary generalized tonic-clonic seizures during **C3**-**C4** seizure period; Case 4: **D1** Background bilateral occipital region 7–8 Hz low to medium wave amplitude mixed Rhythm; During the interval between **D2** attacks, both sides of the bilateral frontal pole, frontal, midline regions showed Intermediate-high amplitude spikes

Routine blood routine, liver and kidney function, thyroid function, blood ammonia, lactate, and other tests of 4 cases showed no significant abnormalities. Various levels of spike and slow spike waves were detected in 24-hour video electroencephalography (Fig. 1). Four cases showed no significant abnormalities in their cranial MRI (3.0T) examinations.

### Gesell developmental progress score

Gross motor DQ, fine motor DQ, adaptive behavior DQ, language DQ, and personal social DQ for nervous system examination, the measured results are expressed in quotient form as developmental quotient (DQ), with DQ = developmental age/actual age x 100. Case 1 and Case 3 have significantly delayed developmental quotient, exhibiting moderate developmental delay, while Case 2 and Case 4 have mild developmental delay (Table 2). As of the end of follow-up, the motor development quotient of the 4 cases has significantly improved compared to before.

**Table 2:** The Gesell developmental schedule score was utilized to assess four pediatric patients with epilepsy resulting from CNSK2B gene variants

Cases	Gross motor skill DQ	Fine motor skill DQ	Adaptive behavior DQ	Language skill DQ	Personal-Social Behavior DQ	Overall assessment DQ
1	78	75	48	48	72	68
2	80	80	76	76	79	79
3	77	72	60	60	70	69
4	82	80	66	66	75	76

*Abbreviations*: DQ Developmental quotient

### Genetic testing results

**Fig. 2:**
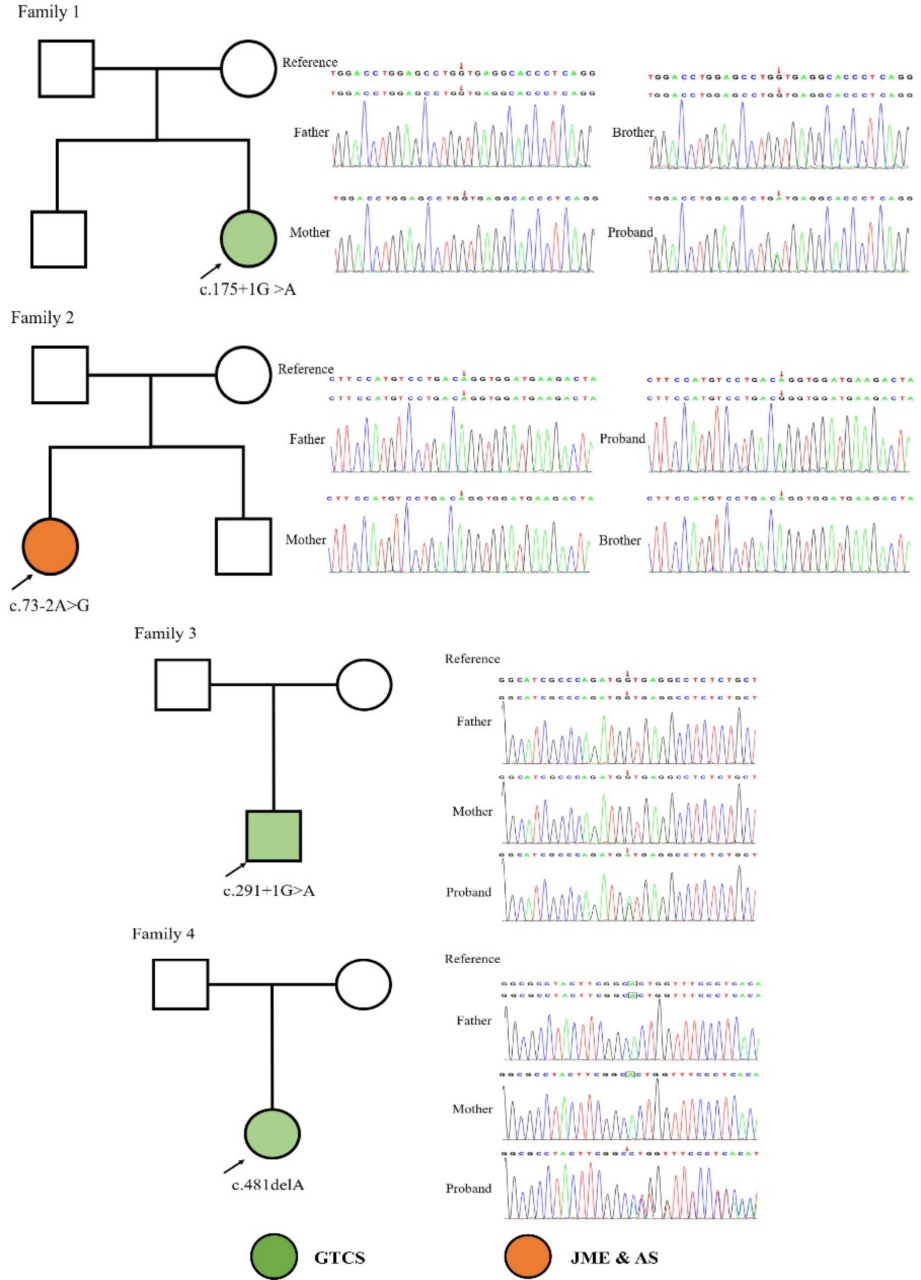
Gene sequencing of four case and their families with Poirier Bienvenu neurodevelopmental syndrome related to CSNK2B gene variant. Family 1 (Case 1): c.175 + 1G > A splicing mutation (arrow) in CSNK2B gene was detected, and both parents and brother are wild-type; Family 2 (Case 2): c.73–2 A > G splicing variant (arrow) in CSNK2B gene was detected. the parents and younger brother are wild-type; Family 3 (Case 3): c.291 + 1G > A splicing variant (arrow) in CSNK2B gene was detected, and the parents are wild-type; Family 4 (Case 4): c.481delA frameshift variant (arrow) in CSNK2B gene was detected, and the parents are wild-type. GTCS: generalized tonic-clonic seizure, JME: positive myoclonic seizure, AS: absence seizure

**Fig. 3:**
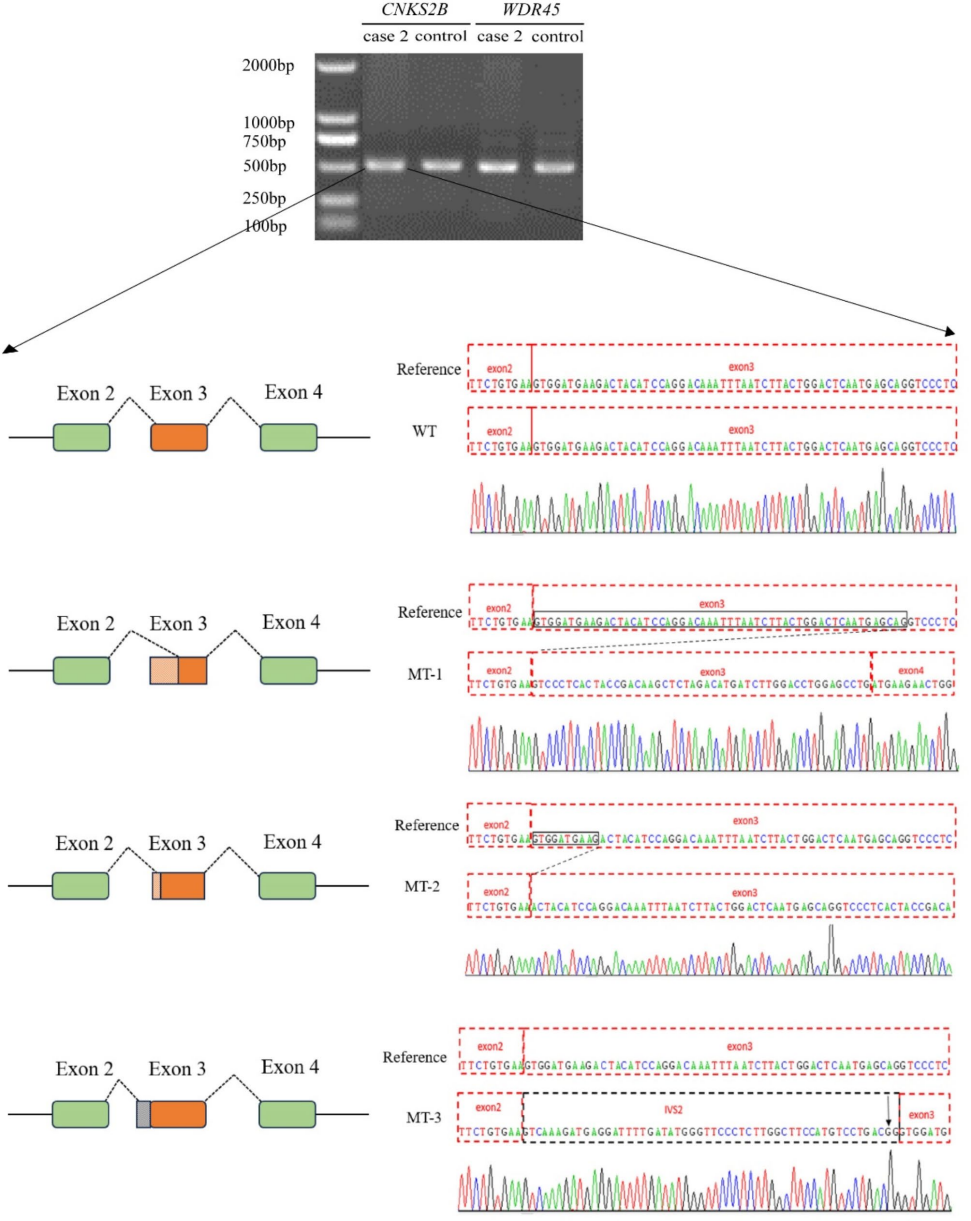
Case 2 of reverse transcription quantitative PCR analysis: Three different *CSNK2B* transcripts caused by c.73–2 A > G variant in *CSNK2B*, namely wild-type (WT) and mutation-type (MT) variant 1–3. Among them, MT-1 experienced a deletion of the exon3 5 ‘end 54 bp sequence, resulting in a loss of 18 amino acids compared to the original amino acid after the variant. MT-2 experienced a deletion of the exon3 5 ‘end 10 bp sequence, leading to premature termination of the amino acid sequence. After the deletion, it encoded 46 amino acids. MT-3 results in premature termination of amino acid coding due to the retention of the 54 bp sequence at the 3 ‘end of intron 2, and encodes 40 amino acids after deletion

Four de novo *CSNK2B* gene heterozygous variants were detected by trio-WES in 4 cases, including c.175 + 1G > A, c.73-2A > G, c.291 + 1G > A splicing variant, and c.481delA frameshift variant, as shown in (Fig. 2). Among them, c.175 + 1G > A, c.291 + 1G > A and c.481delA, are novel variants. Reverse transcription PCR analysis showed that in case 2, *CSNK2B* c.73-2A > G leaded to either exon3 5 ‘end partial sequence deletion or intro2 3’ end partial sequence retention (Fig. 3 and Supplementary Figure). The four variants are not included in the 1,000 genomes, gnomAD, and ExAC databases.

### Treatment plan and follow up situation

Case 1 Early oral administration of levetiracetam and oxcarbazepine controlled for 2 years without recurrence, and later recurrence with the addition of clonazepam, the effect was unsatisfactory. The remaining 3 cases were treated with oral sodium valproate oral solution to stop convulsions, effectively controlling the seizures. No more seizures occurred after oral administration of the medication. Four cases with milestone developmental delay were accompanied by varying degrees of intellectual, language, and cognitive developmental disorders, and all received rehabilitation treatment. After 1–2 years of follow-up, follow-up revealed significant improvements in fine motor skills, gross motor skills, and social adaptability compared to before. However, language recovery was slower and there were varying degrees of language disorders.

## Discussion

At present, it has been found that the *CSNK2B* variant leads to significant differences in epilepsy phenotypes in POBINDS cases So far, there have been no reports of *CSNK2B* related cases dying from seizures, indicating a lower risk of mortality from *CSNK2B* related epilepsy. In this study, three cases were treated with sodium valproate (20 mg/kg.d) starting with treatment, and the seizure symptoms were well controlled after medication. In clinical practice, corresponding measures such as rehabilitation therapy are also given for the symptoms of neurodevelopmental delay. After 3–6 courses of treatment, a psychological and behavioral evaluation of the child was conducted. The evaluation found that after rehabilitation, the child’s large motor and fine motor movements recovered in the same age group, but the language recovery was slower, significantly lagging behind that of the same age group. There are certain differences in the treatment outcomes of epilepsy among patient with POBINDS due to different clinical manifestations. The reported patients of POBINDS indicate that *CSNK2B* related epilepsy responds relatively well to VPA. In two previous case cohorts, patients with intractable epilepsy had more severe neurologic impairment [2, 3]. We report that case 1, despite treatment with multiple antiseizure medication, was still uncontrolled and had more severe neurodevelopment disorders than the case 2–4. So far, the mechanism of *CSNK2B* variant causing epilepsy is not clear. The concurrent use of multiple anti-seizure medications (ASMs) is prevalent, and these medications possess distinct antiseizure mechanisms. Consequently, it becomes challenging to precisely identify the specific target of antiseizure medication. Although more than half of cases have controlled seizures, larger scale prospective studies are still needed to determine more effective treatment methods.

A neurodevelopmental phenotype was previously reported in 24 out of 25 individuals [2]. Similarly, four of our pediatric cases demonstrated varying degrees of developmental delay. However, a recent study reported that three out of nine subjects developed within normal limits. This discrepancy may be attributed to the younger age of participants, as cases in their study were aged two years or younger. Mild intellectual disabilities and learning disabilities may only manifest later, so the incidence of mild intellectual disabilities may be underestimated.

There are literature reports that the activity of CK2 is related to myogenic and osteogenic differentiation [13], which may explain the endocrine phenotype of POBINDS patients. Symptomatic supportive treatment with growth hormone (GH) was given, and the symptoms improved significantly [10, 14–17]. This study identified that case 2 exhibited growth retardation, a finding consistent with the previously reported six cases. This further substantiates that short stature is a variable phenotype associated with POBINDS, potentially linked to aberrant CK2 activity [18]. Furthermore, Case 3 exhibited a blunted facial expression and micropenis. Case 4 demonstrated irregular white spots on the left thigh. For Case 2, during her most recent follow-up examination, uterine ultrasonography revealed multiple small follicles bilaterally in the ovaries as well as a minor pelvic fluid collection. These phenotypes require validation through extended follow-up periods and a larger cohort of case.

The splicing variant c.73–2 A > G affects the consensus site of the 5 ‘GT splicing donor. However, due to the complete reading of introns, these variants may also lead to the production of abnormal transcripts and the use of recessive splicing donor sites. In this study, we identified aberrant transcripts under both conditions. Reverse transcription was conducted on case 2, revealing that the mutated transcript was missing exon 1 (18 nucleotides), the transcript was missing exon 2 (46 nucleotides), and he transcript was missing exon 3 (40 nucleotides). Exon deletion, insertion stop codon, abnormal proteins are produced, and the C-terminus of the protein, including the metal binding site and the interaction domain with CSNK2A, should be completely lost, indicating that these variants have loss the function.

## Conclusions

There is currently no clear and effective treatment plan for POBINDS, and symptomatic treatment support is the main approach. There are different literature reports that administering different types and doses of ASMs brings different therapeutic effects. In case 1, despite the use of a combination of multiple ASMs, seizure control remained unattainable.

In summary, we presented the clinical phenotypes of four unrelated case with novel heterozygous variants in the *CSNK2B* gene and expands the spectrum of variants in Poirier Bienvenu neurodevelopmental syndrome. Providing a foundation for further investigation into the correlation between genotype and phenotype.

## Electronic supplementary material

Below is the link to the electronic supplementary material.

Supplementary Material 1

Supplementary Material 2

Supplementary Material 3

## Data Availability

Data is provided within the manuscript or supplementary information files”. Find some help on our Data availability statements page.

## Abbreviations

POBINDS: Poirier-Bienvenu neurodevelopmental syndrome
MRI: Magnetic resonance imaging
Trio-WES: Trio Whole Exome Sequencing
DQ: Developmental quotient
ACMG: American Society for Medical Genetics and Genomics
GTCS: Generalized Tonic-Clonic Seizure
JME: Positive Myoclonic Seizure
AS: Absence Seizure
WT: Wild-Type
MT: Mutation-Type
P: Pathogenic
ASM: Anti-Seizure Medication
LEV: Levetiracetam
OXC: Oxcarbazepine
CZP: Clonazepam
VPA: Sodium Valproate
