# Supplementary figures and images for: Genetic analysis of four cases of Poirier Bienvenu neurodevelopmental syndrome associated with CSNK2B variant
Source: BMC Med Genomics. 2025 Apr 10;18:68. doi: 10.1186/s12920-025-02132-5 (PMC11983931; doi:10.1186/s12920-025-02132-5)

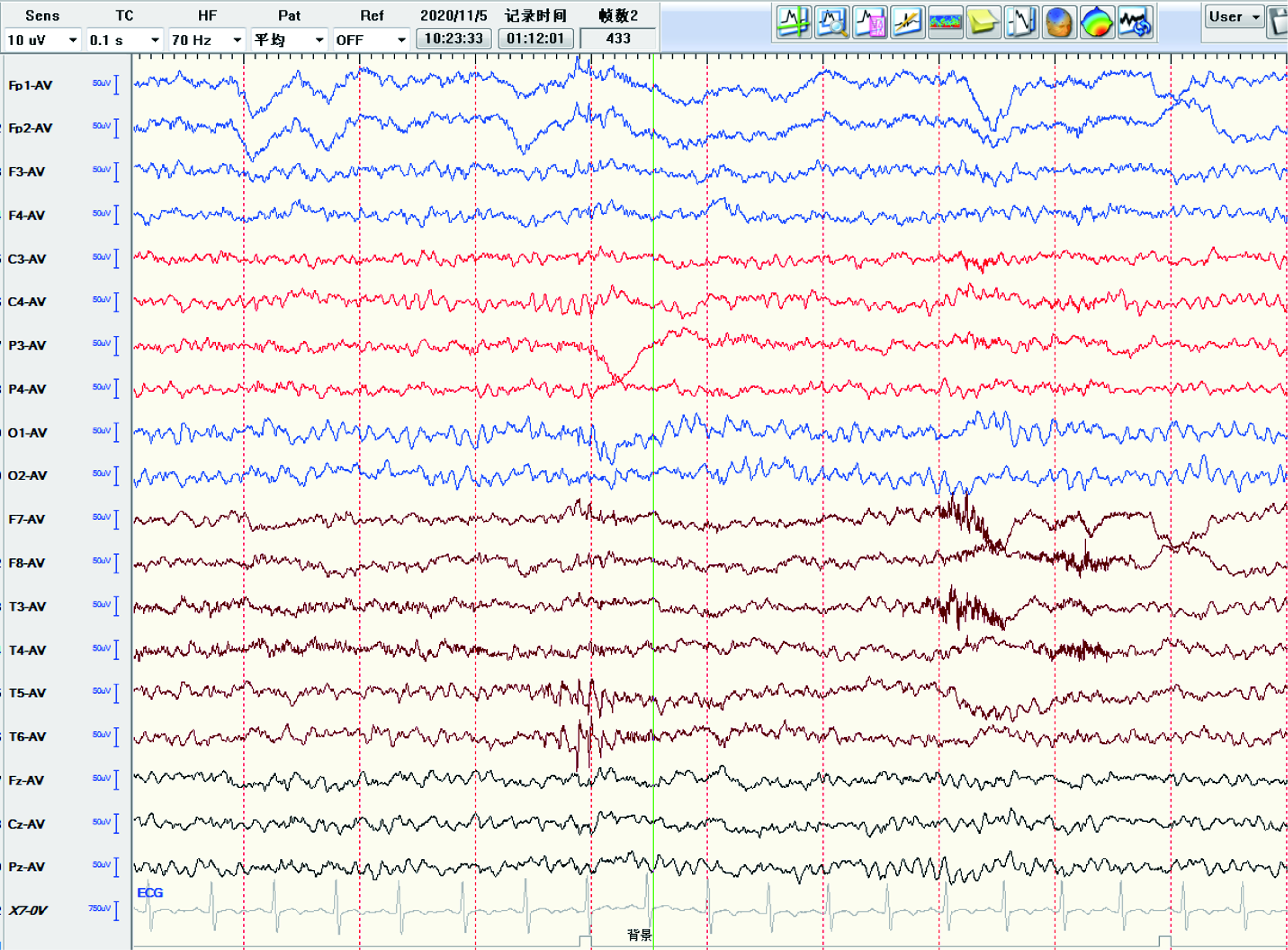

Supplement: Supplementary file 1 — Supplementary Material 1 [file 12920_2025_2132_MOESM1_ESM.zip › Electroencephalogram (EEG) recordings were acquired from four cases/A1.tif]

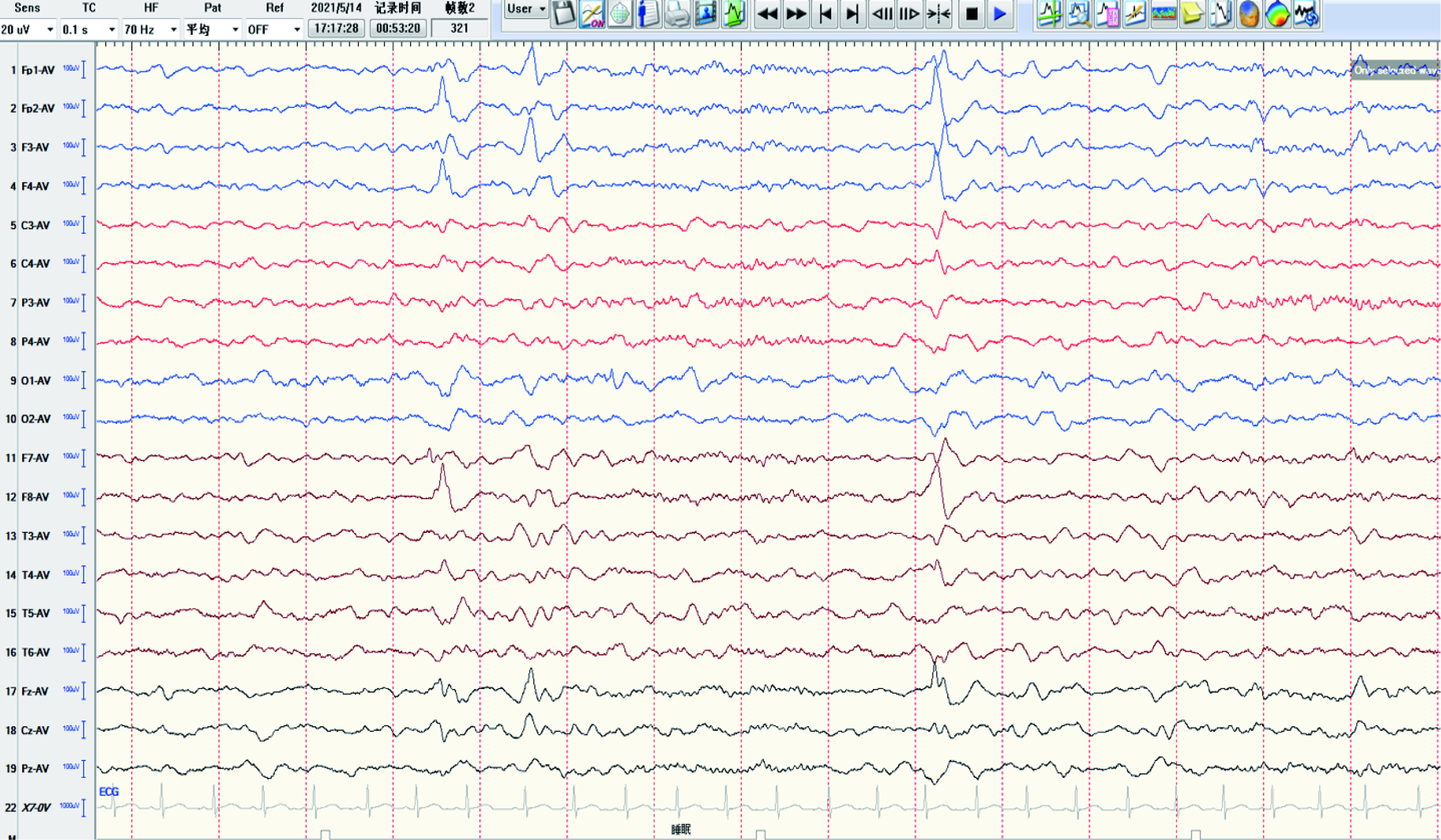

Supplement: Supplementary file 1 — Supplementary Material 1 [file 12920_2025_2132_MOESM1_ESM.zip › Electroencephalogram (EEG) recordings were acquired from four cases/A2.tif]

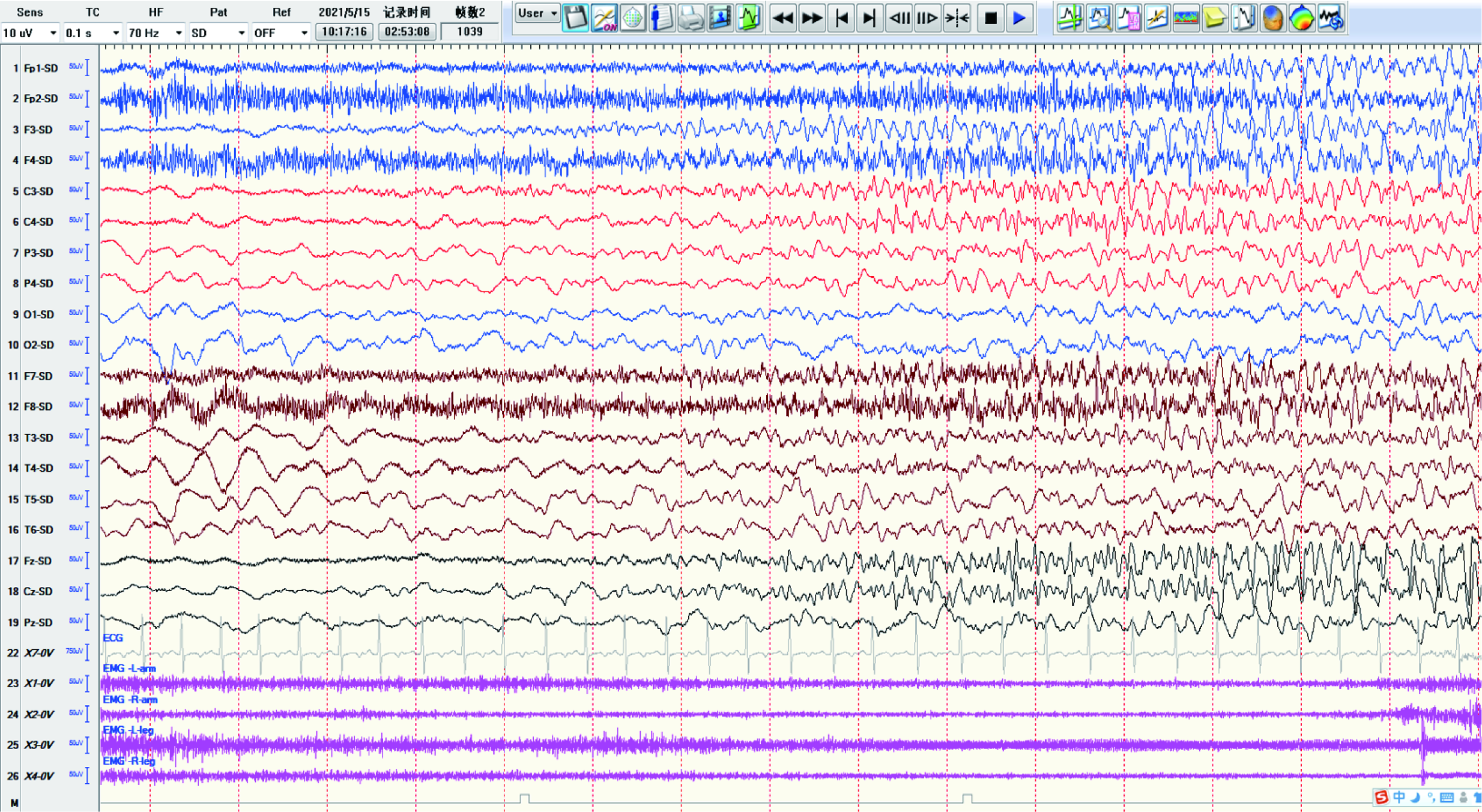

Supplement: Supplementary file 1 — Supplementary Material 1 [file 12920_2025_2132_MOESM1_ESM.zip › Electroencephalogram (EEG) recordings were acquired from four cases/A3.tif]

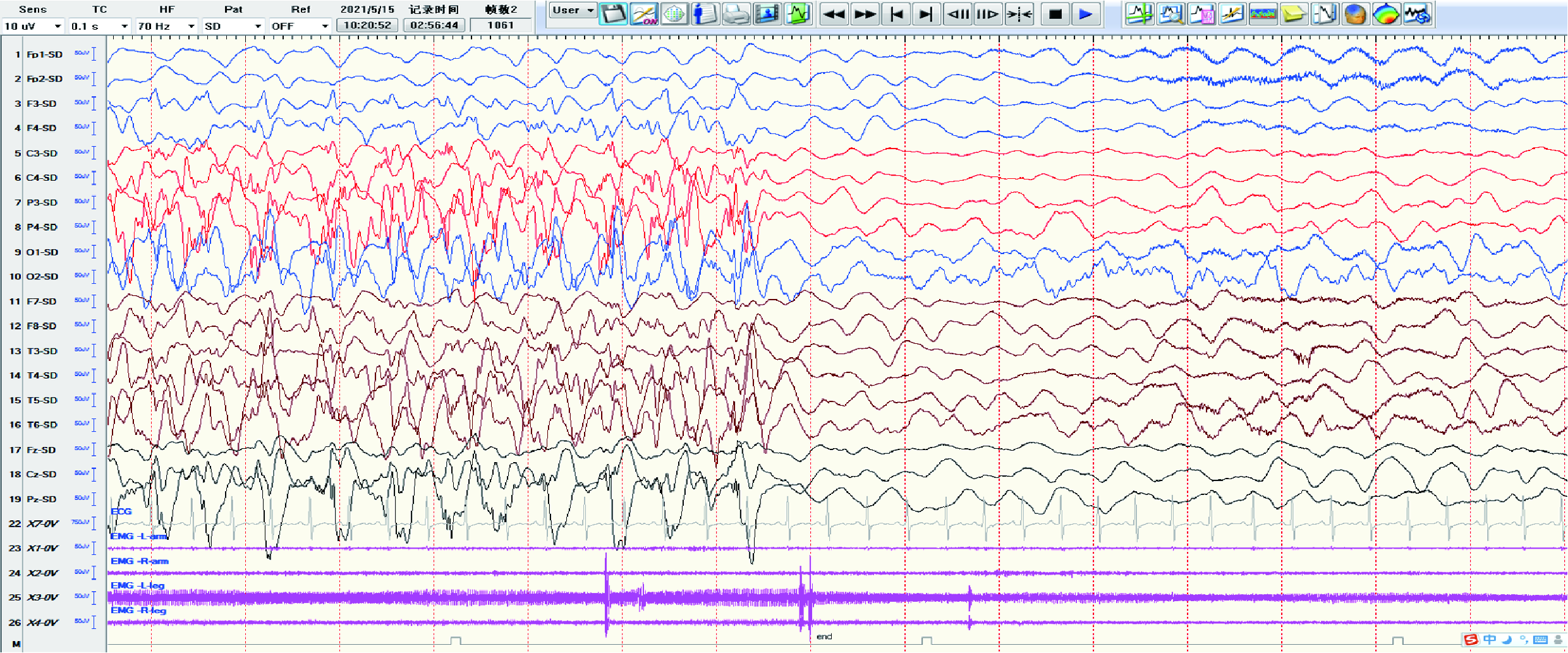

Supplement: Supplementary file 1 — Supplementary Material 1 [file 12920_2025_2132_MOESM1_ESM.zip › Electroencephalogram (EEG) recordings were acquired from four cases/A4.tif]

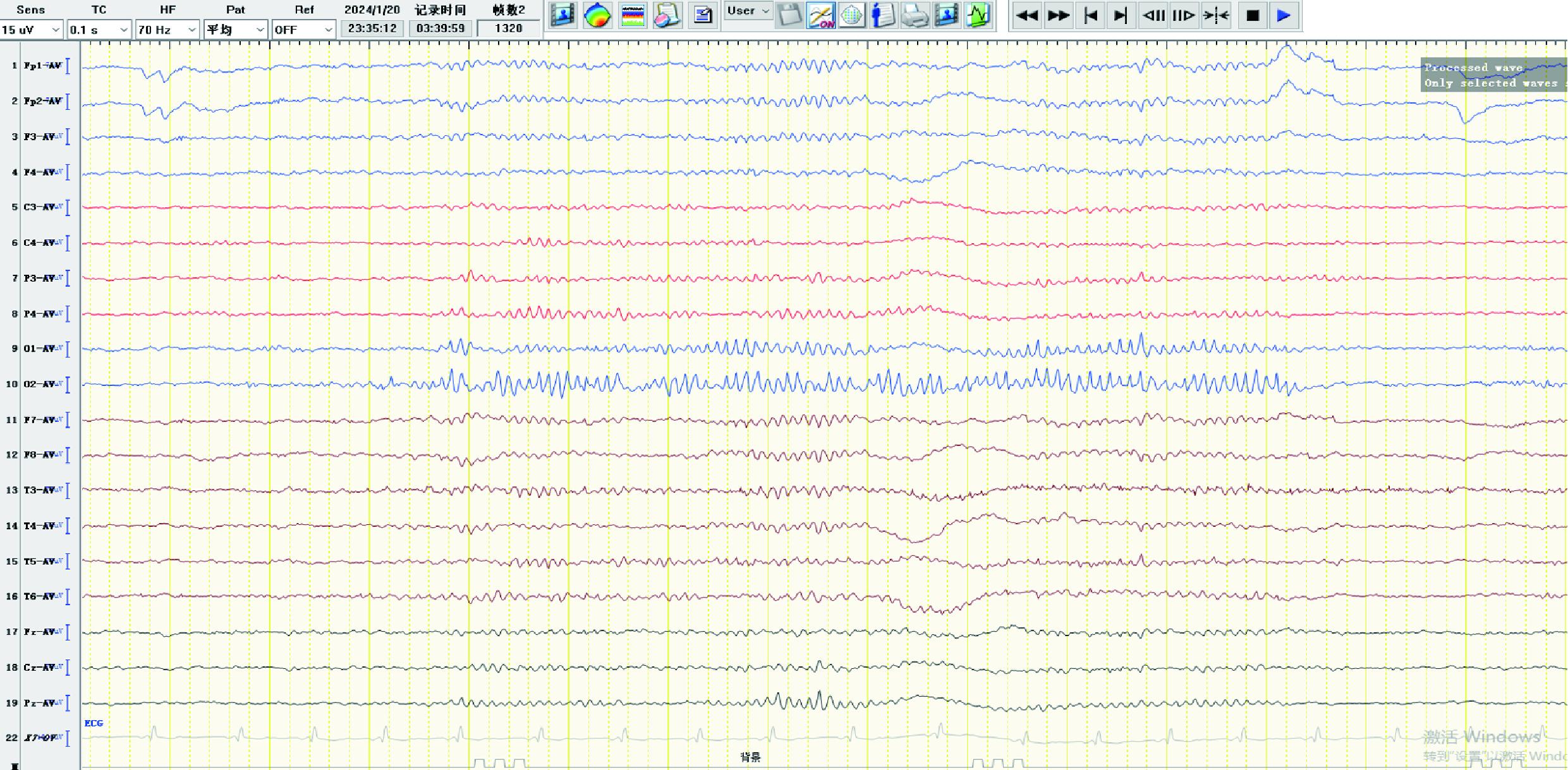

Supplement: Supplementary file 1 — Supplementary Material 1 [file 12920_2025_2132_MOESM1_ESM.zip › Electroencephalogram (EEG) recordings were acquired from four cases/B1.tif]

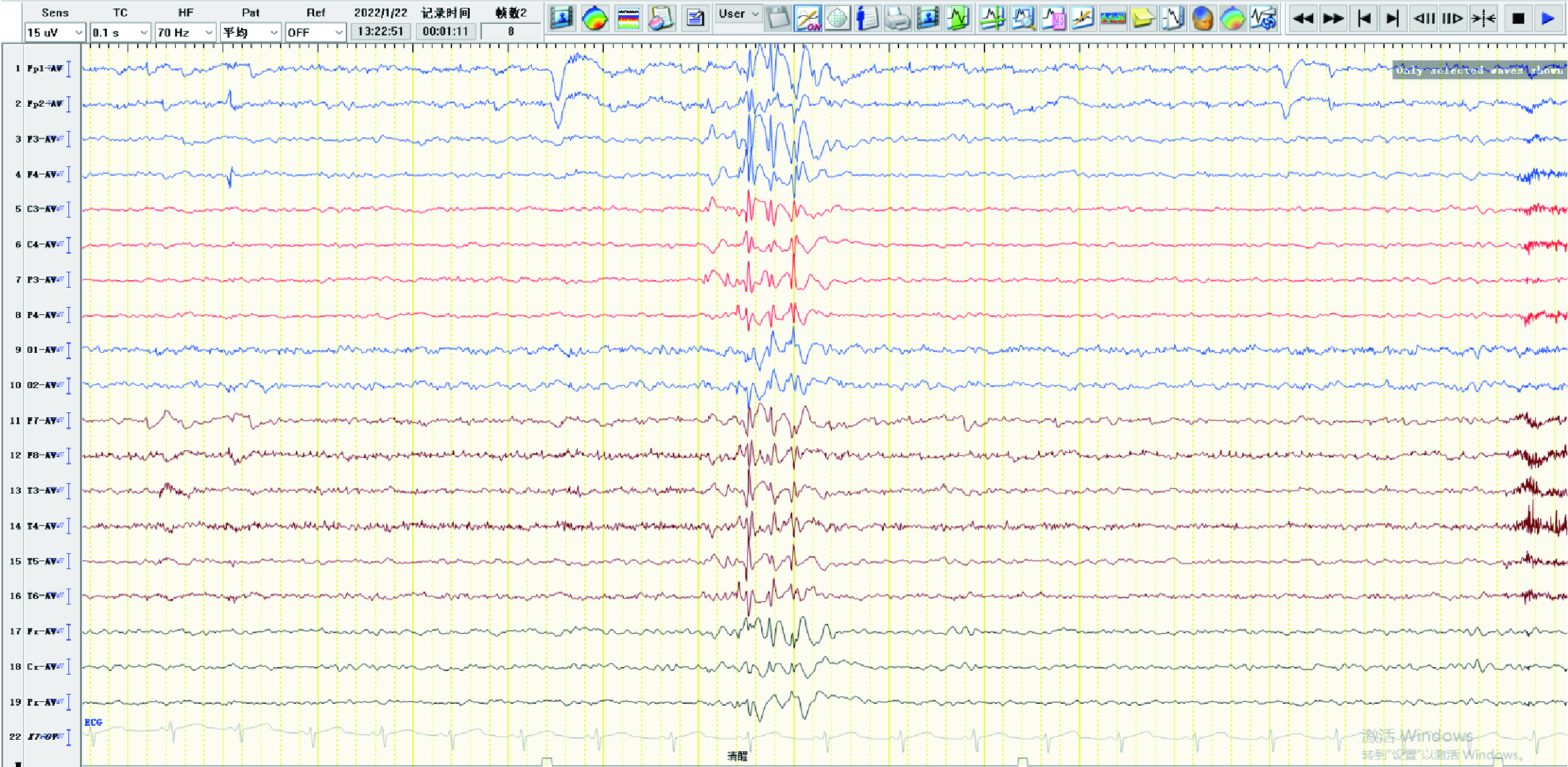

Supplement: Supplementary file 1 — Supplementary Material 1 [file 12920_2025_2132_MOESM1_ESM.zip › Electroencephalogram (EEG) recordings were acquired from four cases/B2.tif]

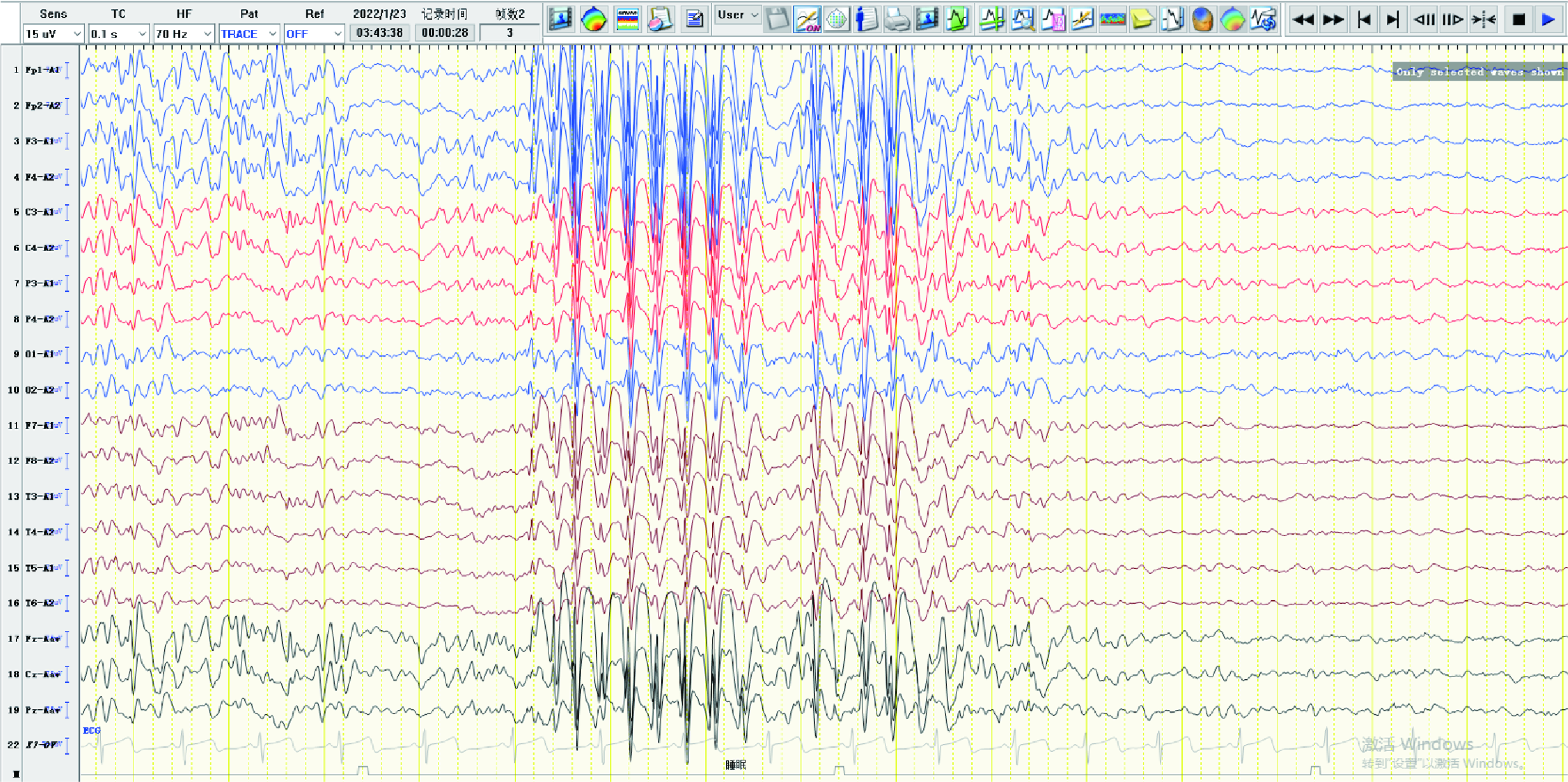

Supplement: Supplementary file 1 — Supplementary Material 1 [file 12920_2025_2132_MOESM1_ESM.zip › Electroencephalogram (EEG) recordings were acquired from four cases/B3.tif]

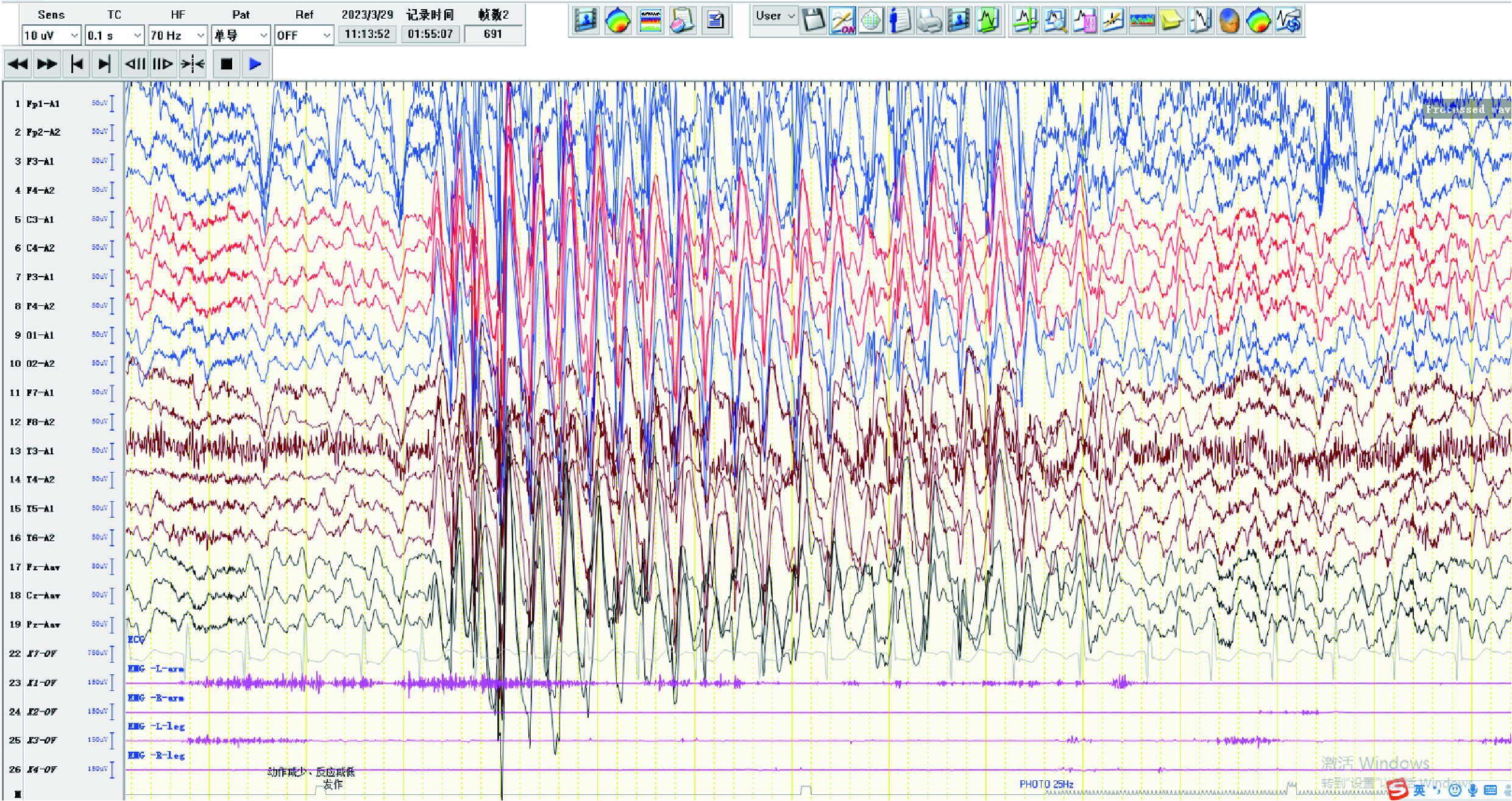

Supplement: Supplementary file 1 — Supplementary Material 1 [file 12920_2025_2132_MOESM1_ESM.zip › Electroencephalogram (EEG) recordings were acquired from four cases/B4.tif]

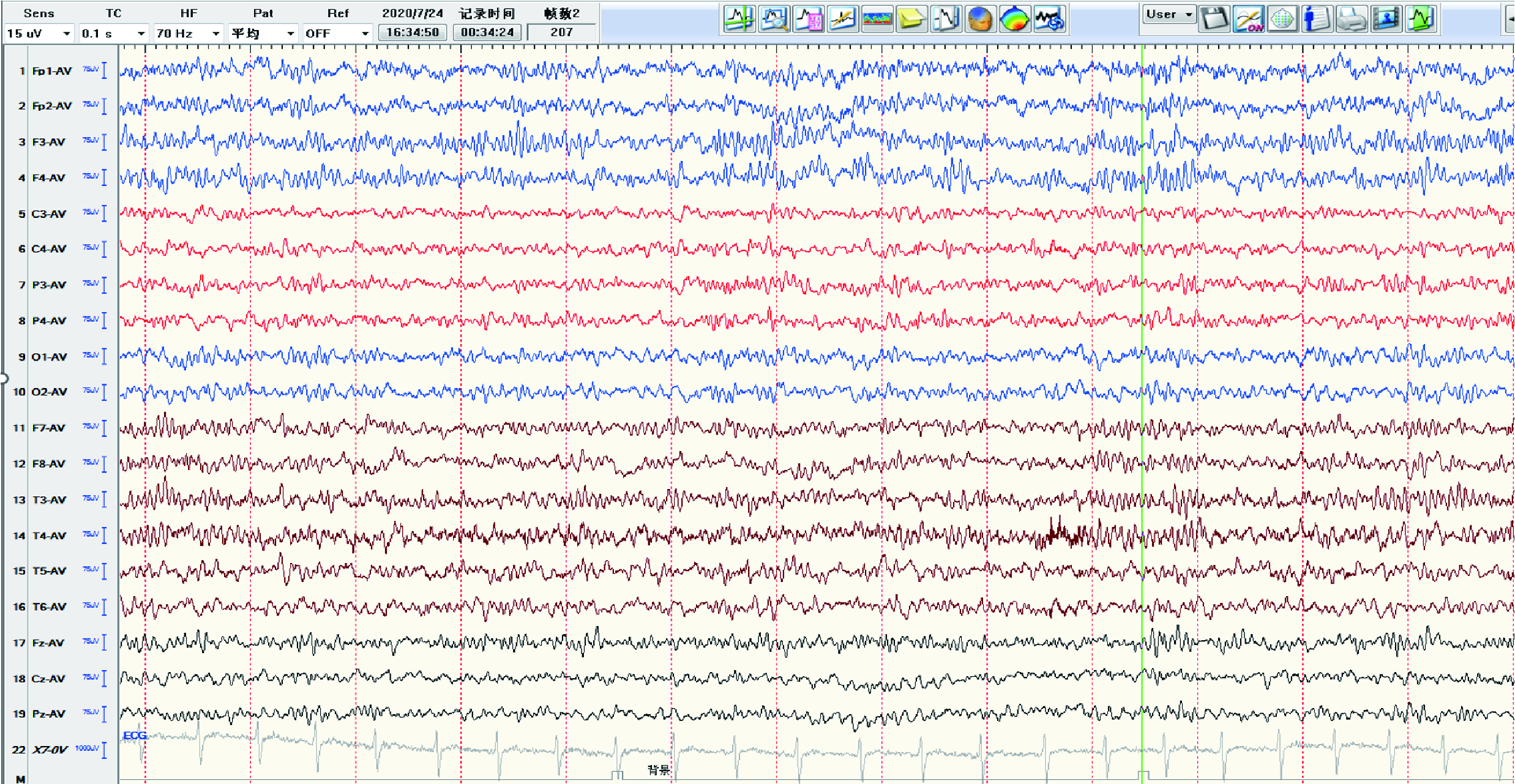

Supplement: Supplementary file 1 — Supplementary Material 1 [file 12920_2025_2132_MOESM1_ESM.zip › Electroencephalogram (EEG) recordings were acquired from four cases/C1.tif]

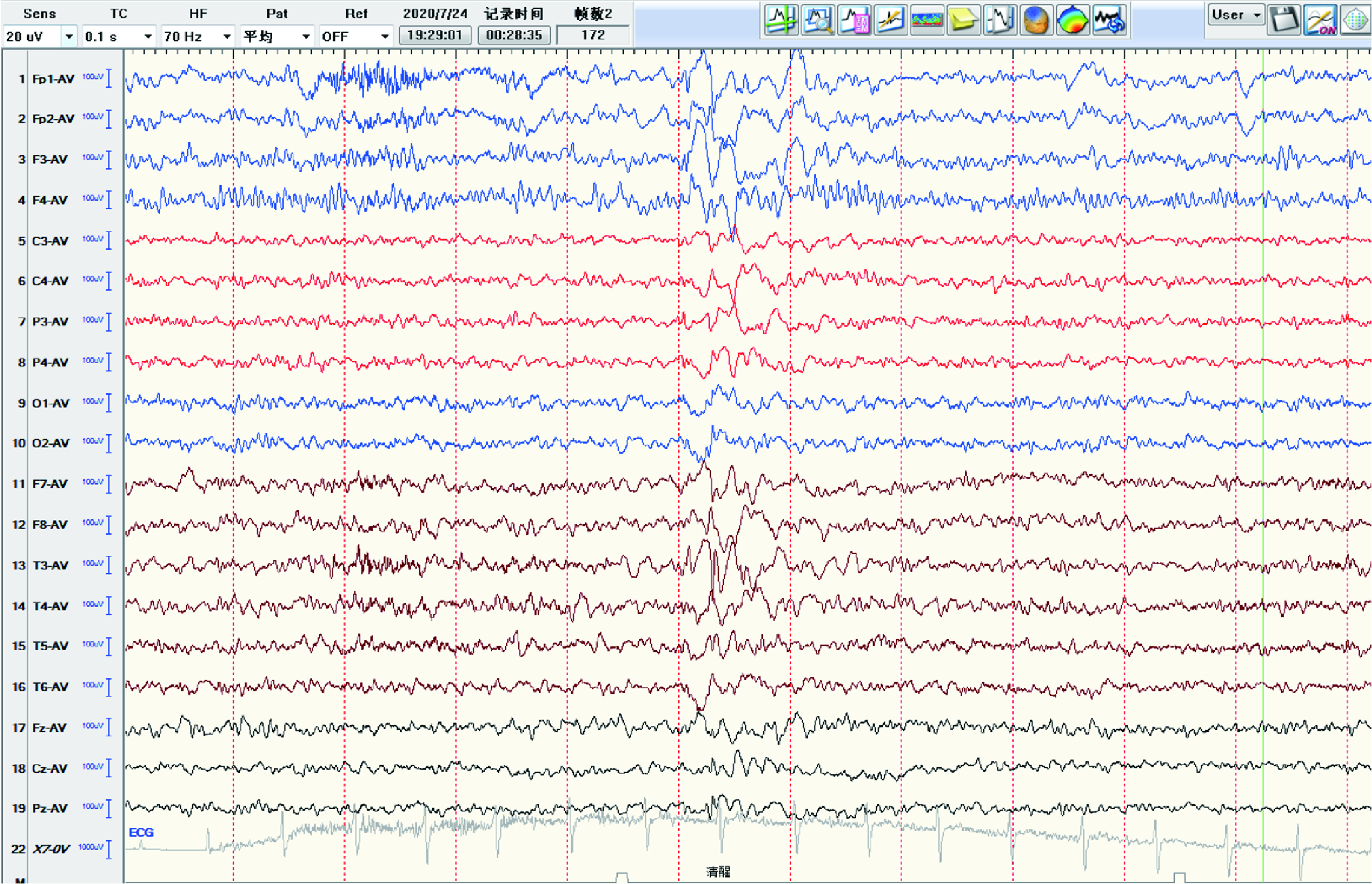

Supplement: Supplementary file 1 — Supplementary Material 1 [file 12920_2025_2132_MOESM1_ESM.zip › Electroencephalogram (EEG) recordings were acquired from four cases/C2.tif]

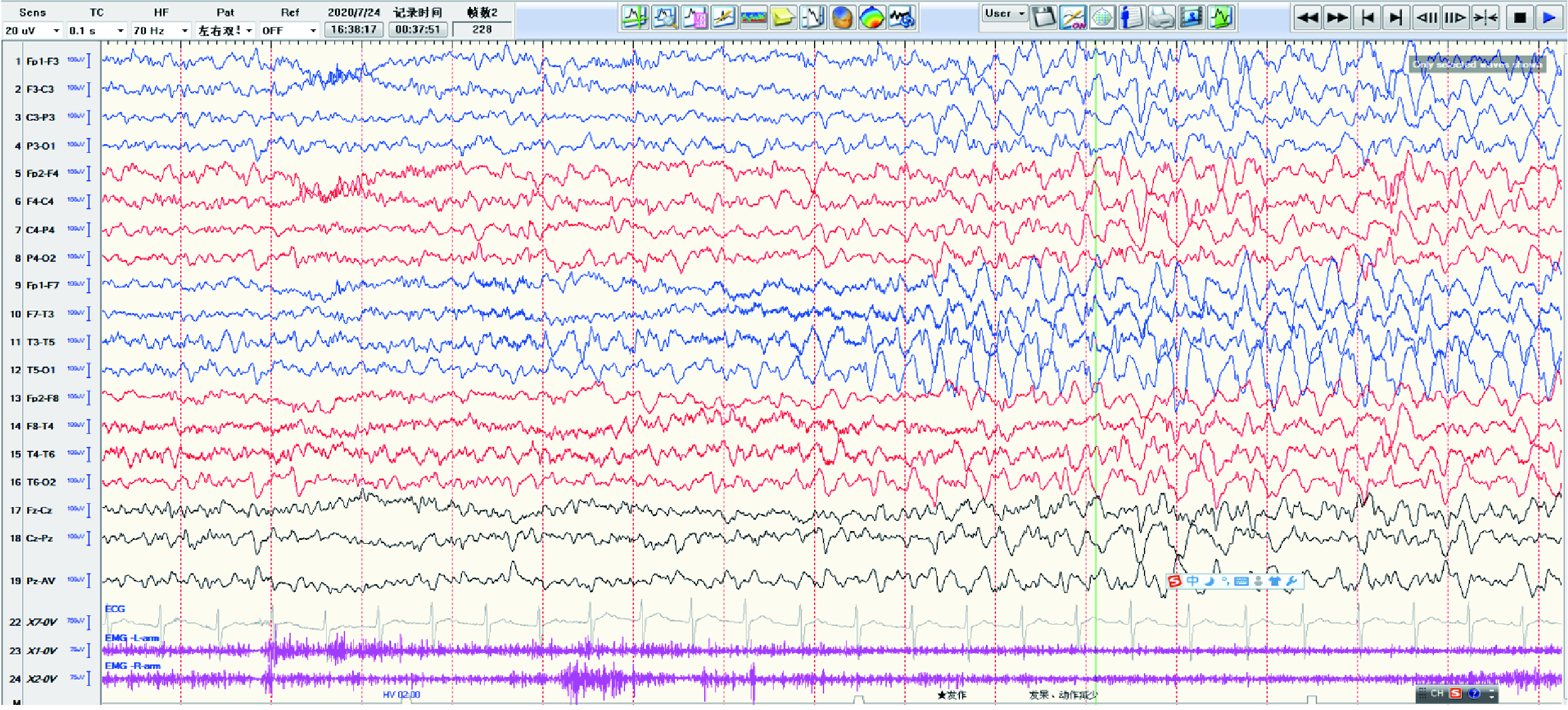

Supplement: Supplementary file 1 — Supplementary Material 1 [file 12920_2025_2132_MOESM1_ESM.zip › Electroencephalogram (EEG) recordings were acquired from four cases/C3.tif]

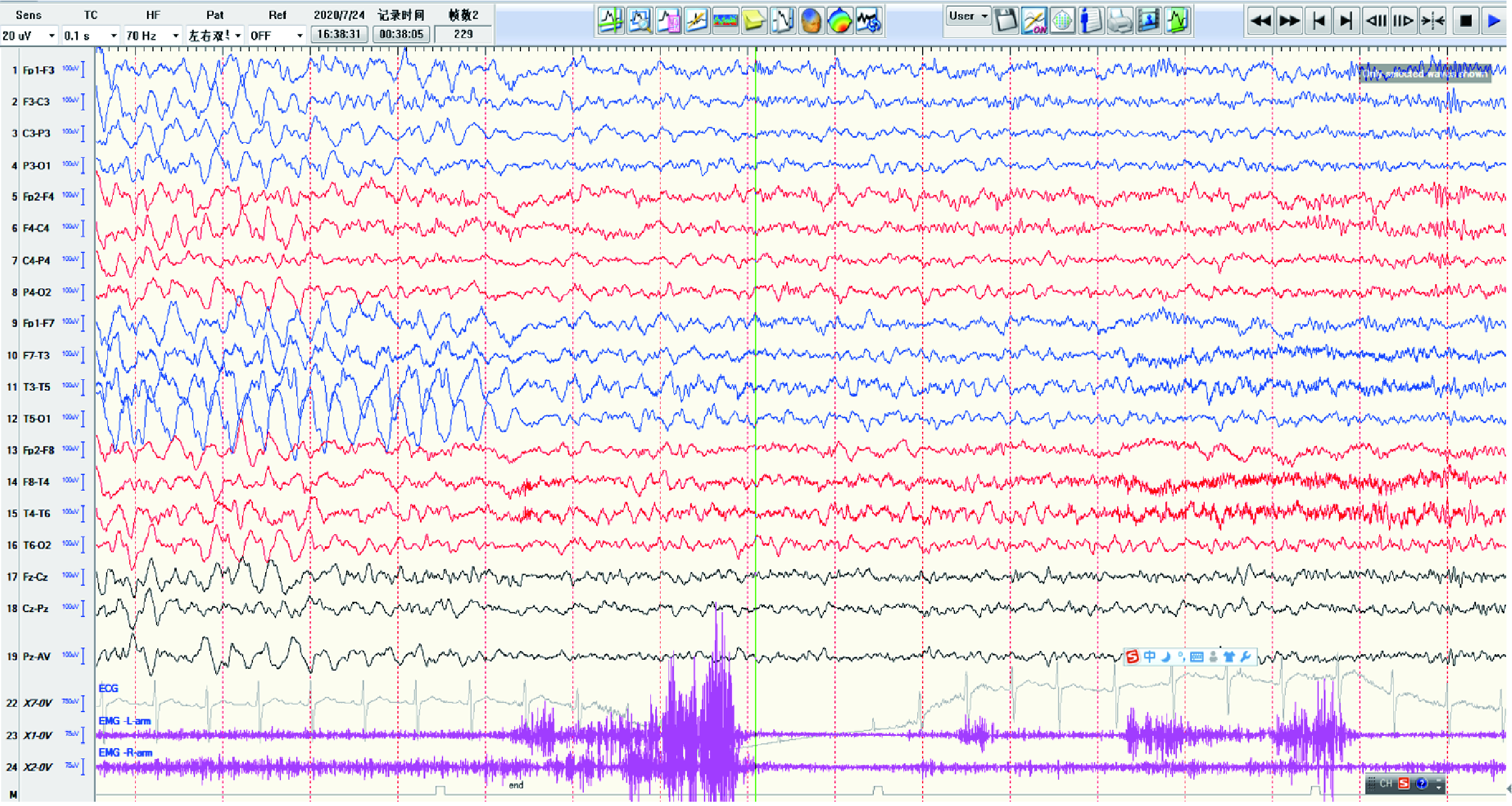

Supplement: Supplementary file 1 — Supplementary Material 1 [file 12920_2025_2132_MOESM1_ESM.zip › Electroencephalogram (EEG) recordings were acquired from four cases/C4.tif]

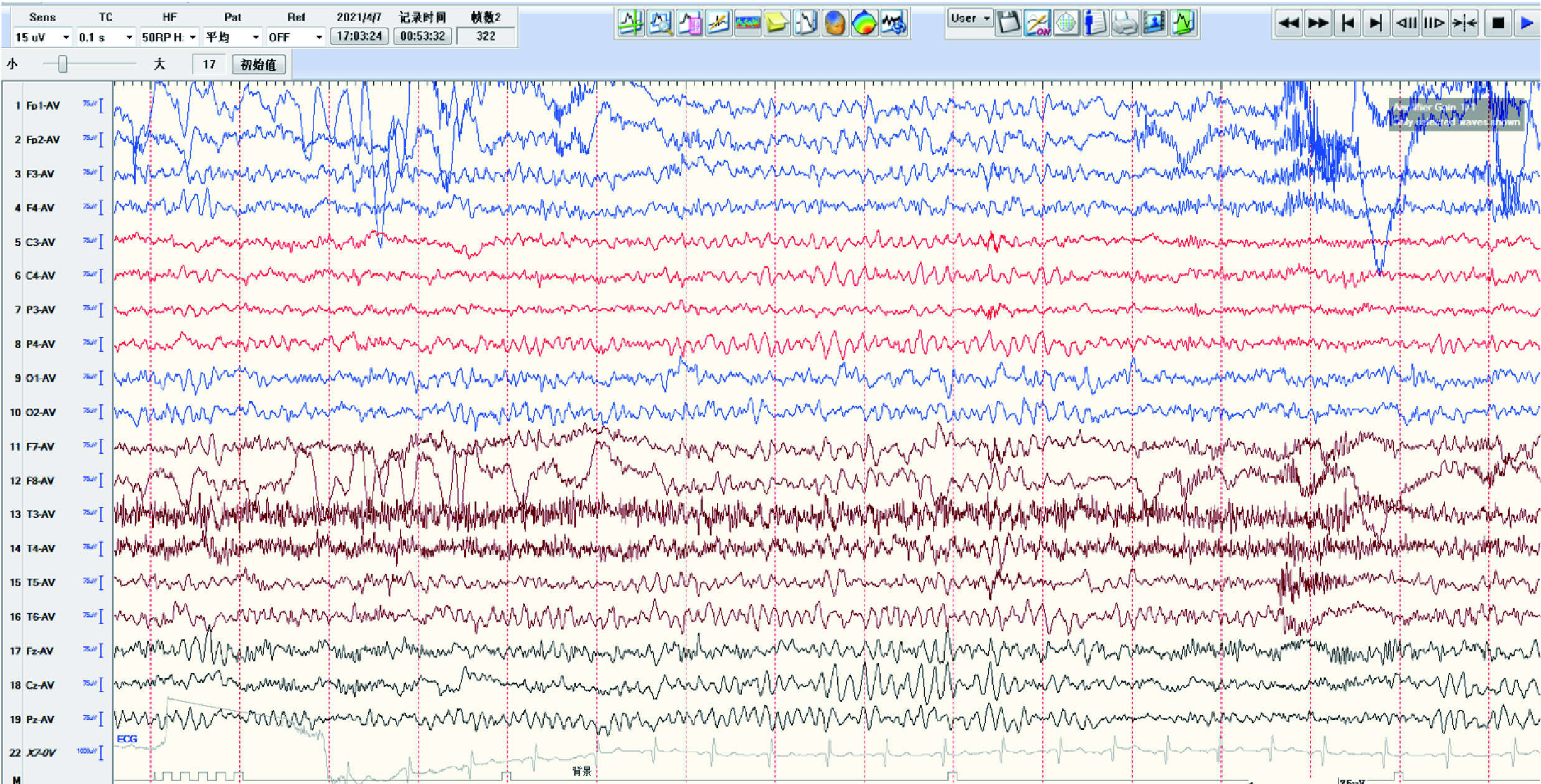

Supplement: Supplementary file 1 — Supplementary Material 1 [file 12920_2025_2132_MOESM1_ESM.zip › Electroencephalogram (EEG) recordings were acquired from four cases/D1.tif]

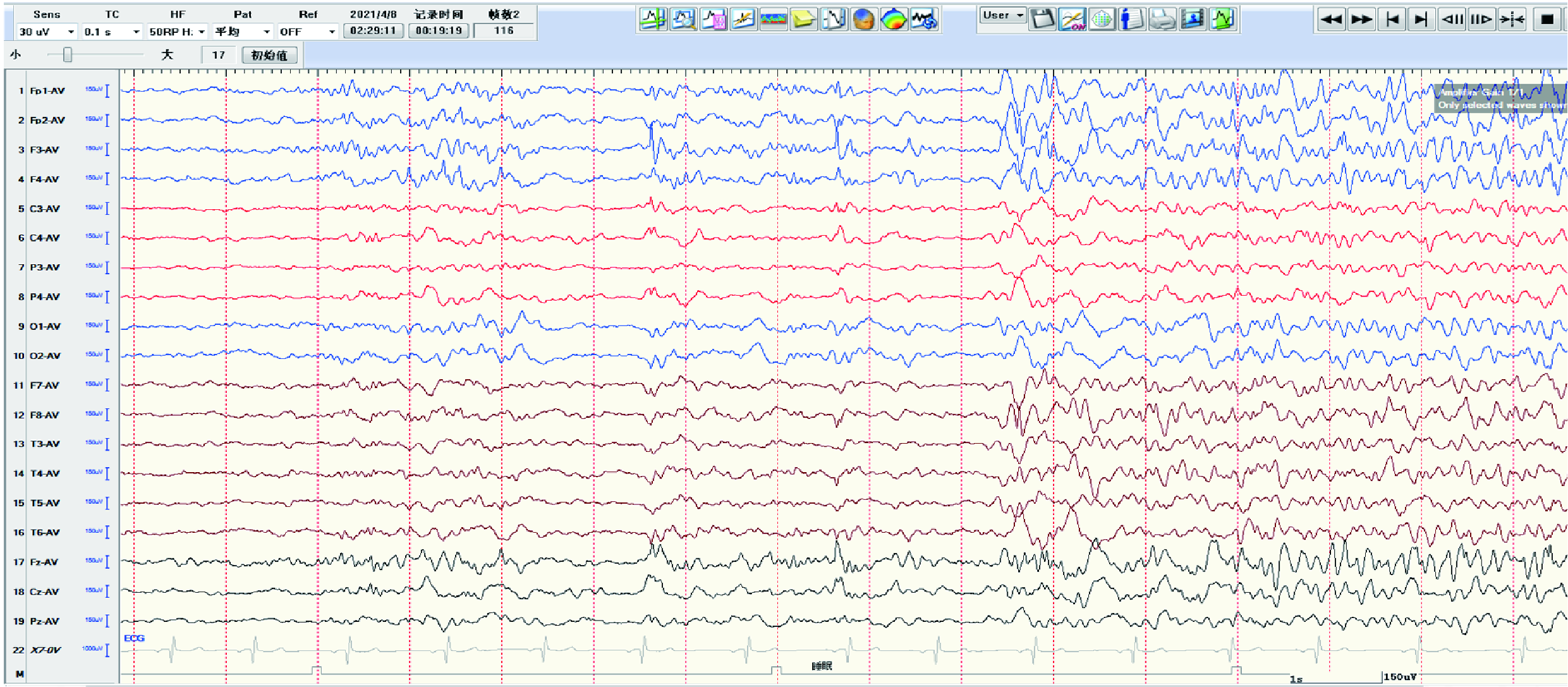

Supplement: Supplementary file 1 — Supplementary Material 1 [file 12920_2025_2132_MOESM1_ESM.zip › Electroencephalogram (EEG) recordings were acquired from four cases/D2.tif]

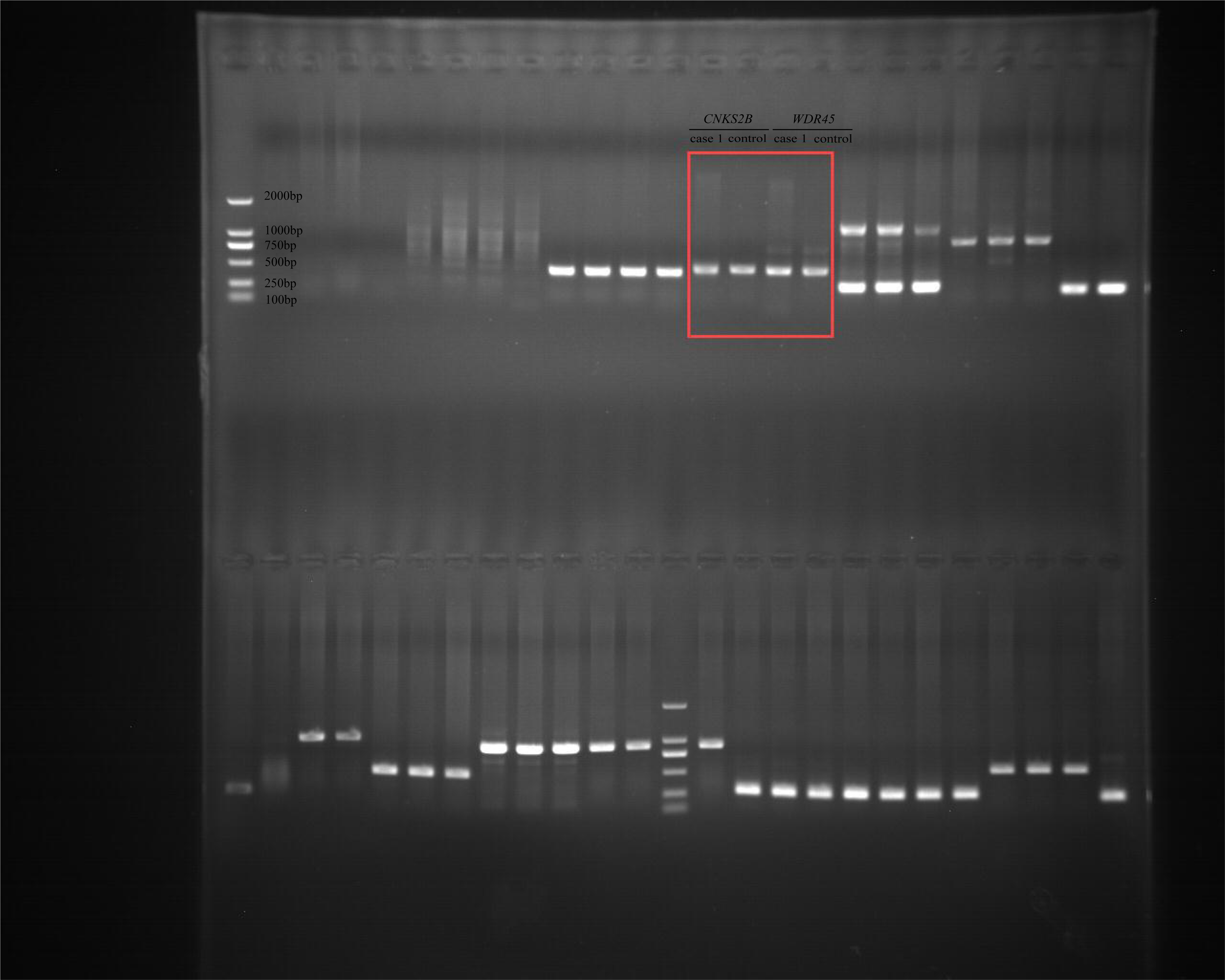

Supplement: Supplementary file 3 — Supplementary Material 3 [file 12920_2025_2132_MOESM3_ESM.jpg]
